# Cultures as types and the utility of viable specimens for fungal nomenclature

**DOI:** 10.1186/s43008-024-00155-8

**Published:** 2024-07-24

**Authors:** Andrey Yurkov, Cobus M. Visagie, Pedro W. Crous, Akira Hashimoto, Christiane Baschien, Dominik Begerow, Martin Kemler, Nathan Schoutteten, Marc Stadler, Nalin N. Wijayawardene, Kevin D. Hyde, Ning Zhang, Teun Boekhout, ICTF Yeast Working Group, Andrey Yurkov, Teun Boekhout, Feng-Yan Bai, Dominik Begerow, Neža Čadež, Heide-Marie Daniel, Jack W. Fell, Marizeth Groenewald, Marc-André Lachance, Diego Libkind, Gábor Péter, Masako Takashima, Benedetta Turchetti, Tom W. May, Marco Thines, David L. Hawksworth

**Affiliations:** 1 Department of Bioresources for Bioeconomy and Health Research, Leibniz Institute DSMZ-German Collection of Microorganisms and Cell Cultures, Brunswick, Germany; 2 Department of Biochemistry, Genetics and Microbiology, Forestry and Agricultural Biotechnology Institute (FABI), University of Pretoria, Pretoria, South Africa; 3 Westerdijk Fungal Biodiversity Institute, Utrecht, The Netherlands; 4 Microbiology, Department of Biology, Utrecht University, Utrecht, The Netherlands; 5 RIKEN BioResource Research Center, Tsukuba, Japan; 6 Organismic Botany and Mycology, Institute of Plant Science and Microbiology, Faculty of Mathematics, Informatics and Natural Sciences, University of Hamburg, Hamburg, Germany; 7 Microbial Drugs, Helmholtz Centre for Infection Research, Brunswick, Germany; 8 Center for Yunnan Plateau Biological Resources Protection and Utilization, College of Biological Resource and Food Engineering, Qujing Normal University, Qujing, Yunnan People’s Republic of China; 9 Tropical Microbiology Research Foundation, Pannipitiya, Sri Lanka; 10 Center of Excellence in Fungal Research, Mae Fah Luang University, Chiang Rai, Thailand; 11 School of Science, Mae Fah Luang University, Chiang Rai, Thailand; 12 Department of Plant Biology, Rutgers University, New Brunswick, NJ USA; 13 Department of Biochemistry and Microbiology, Rutgers University, New Brunswick, NJ USA; 14 College of Science, King Saud University, Riyadh, Saudi Arabia; 15 The Yeasts Foundation, Amsterdam, The Netherlands; 16 IUMS International Commission on Yeasts, Bratislava , Slovakia; 17 Royal Botanic Gardens Victoria, Birdwood Avenue, Melbourne, VIC 3004 Australia; 18 Department of Biological Sciences, Institute of Ecology, Evolution, and Diversity, Goethe University, Frankfurt Am Main, Germany; 19 Senckenberg Biodiversity and Climate Research Centre, Frankfurt Am Main, Germany; 20 Comparative Fungal Biology, Royal Botanic Gardens, Kew, Richmond, Surrey TW9 3AE UK; 21 Department of Life Sciences, Natural History Museum, London, SW7 5BD UK; 22 School of Geography and Environmental Science, University of Southampton, Southampton, SO17 1BJ UK; 23 Jilin Agricultural University, 2888 Xincheng Avenue, Chanchung, China

## Abstract

The debates over the requirement of *the International Code of Nomenclature for algae, fungi, and plants* (ICNafp) for a viable specimen to represent the name-bearing type material for a species or infraspecific taxon have a long history. Taxonomy of fungi commonly studied as living cultures exemplified by yeasts and moulds, strongly depend on viable reference material. The availability of viable cultures is also particularly useful for several groups of filamentous and dimorphic fungi. While the preservation of metabolically inactive cultures is permitted and recommended by the ICNafp, there is room for improvement. Below, we review the history and current status of cultures as the name-bearing type material under the *Code*. We also present a roadmap with tasks to be achieved in order to establish a stable nomenclatural system that properly manages taxa typified by viable specimens. Furthermore, we propose setting up rules and defining the nomenclatural status of ex-type cultures under Chapter F, the section of the ICNafp that includes provisions specific to names of fungi.

## Introduction

The debates over the requirement of the *International Code of Nomenclature for algae, fungi, and plants* (ICNafp or the *Code*, Turland et al. 2018) for a viable specimen to represent the name-bearing type material for a species or infraspecific taxon have a long history. Living material was permitted as a type for all groups covered by the then *International Code of Botanical Nomenclature* (which explicitly covered fungi) up to the Montreal Congress of 1959, when it was then restricted to bacteria and fungi (Montreal *Code*: Lanjouw et al. 1961: Art. 9 Note 3). The issue of how living cultures were treated, and the direction of travel of the provisions of the *Code* led to the split with the microbiologists and the publication of the *International Code of Nomenclature of Bacteria and Viruses* (Buchanan 1959). Living cultures of fungi were first excluded at the Edinburgh Congress in 1964 (Edinburgh *Code*: Lanjouw et al. 1966: Art. 9 Note 3), although they were still permitted for bacteria.

How living type cultures of fungi came to be omitted in 1964 does not seem to have been based on any Congress vote. There was no proposal to remove the provision for type cultures of fungi put to the Edinburgh Congress. Indeed, the decision to omit was evidently based on a comment by M.A. Donk stating “*by an oversight the word Fungi had been kept in contrary to the Montreal decision. It should be deleted*” (Stafleu 1966: 17). There is, however, no record of any debate or exchange on this point occurring at the Edinburgh Congress, and no formal proposal appears to have been made in Montreal (Lanjouw 1959). In debate at the Montreal Congress, however, R.E. Buchanan had requested the Committee for Fungi be asked to consider the recognition of “type cultures”. The Committee report including this provision is recorded as supported by a majority of 5–2 and was referred to the Editorial Committee. That Committee undertook to discuss the matter with old and new members of the Committee for Fungi (Bureau of Nomenclature 1960: 101–105). Excluding lichenologists, there were only some 18 mycologists registered for the Nomenclature Section in Montreal and 20 in Edinburgh, though most did not work with fungi in culture. Despite this unsatisfactory and clearly questionable situation, which had never been discussed by a broad spectrum of mycologists, the issue of type cultures of fungi was not raised at the subsequent Seattle Congress of 1970.

The issue of allowing living cultures of fungi as nomenclatural types was a hot topic at the first International Mycological Congress (IMC1) in Exeter in 1971, just five years after publication of the Edinburgh *Code*. A Nomenclature Secretariat was set up at that Congress with several subcommittees, one of which was devoted to the issue of living types, in which the then Director of the Centraalbureau voor Schimmelcultures (CBS; now the Westerdijk Fungal Biodiversity Institute, WI), Adolf von Arx, was especially keen to reverse the change. One of us (DLH) was a member of that Subcommittee, which reported to IMC2 in Tampa in 1977. This led to proposals being formulated and presented to the International Botanical Congress (IBC) in Yokohama in 1993 (Hawksworth 1993).

Those proposals included allowing “cultures as types” and as a result, the Tokyo *Code* (Greuter et al. 1994) endeavoured to resolve the issue in a manner acceptable to botanists by specifying that types had to be “*preserved permanently and cannot be living plants or cultures*” (Greuter et al. 1994: Art. 8.2). An example was voted into the Code which explained that a culture was acceptable as type material if “*it is permanently preserved in a metabolically inactive state by lyophilization*” (Art. 8.2 Ex. 1). This was the first official recognition of a permanent preservation techniques (e.g., cryopreservation and freeze-drying) being acceptable for the type material of algae and fungi. No date when this was to become effective was specified; this was deliberate as it meant that the rule was retroactive, and that previously lyophilized or liquid nitrogen stored material was now acceptable to serve as name-bearing type material.

Another important modification adopted in the Tokyo *Code* was the recommendation to deposit such material “*in at least two institutional or genetic resource collections*” (Rec. 8B.1) and the use of “ex-” as a prefix to identify resuscitated cultures originating from the metabolically inactive name-bearing type culture (Rec. 8B.2). The only substantive change since that time has been the requirement from 1 Jan. 2019 to include “*a statement that the culture is preserved in a metabolically inactive state”* in order to be validly published, a rule that is not retroactive (Turland et al. 2018: Art. 40.8).

By their very nature, microscopic fungi are commonly isolated, purified, and studied on culture media under controlled laboratory conditions to observe, for example, the life-cycle, as well as for analysing their physiological and molecular properties. Consequently, the investigated material and designated type material ultimately represent viable cultures. A revision of such a taxon involving the nomenclatural type or any other reference material will be based on a revived culture of a holo- or isotype (such a strain is then called an ex-type culture), not the dead type specimen or a metabolically inactive culture that serves as a type. In his proposal, Hawksworth (1993) outlined several practical considerations for “living types”, such as testing for substrate assimilation, growth characteristics, enzymatic properties, secondary metabolite profiles and host specificity tests. Indeed, the availability of such viable cultures that have a nomenclatural status would offer several advantages, including the possibility of propagation for authentication purposes, experiments using new techniques and tools, and exchange of progenies between repositories. Availability of the material from several repositories achieved through exchange between culture collections ensures safe preservation and facilitates access to the material. However, while a type can now be metabolically inactive but viable, any cultures derived from it do not have any formal nomenclatural status. When all types are lost, which is a major impediment to the interpretation of names, ex-type cultures allow for the authentication and revision of species, as these are usually done on ex-types. The ex-type cultures can then be preserved again in a metabolically inactive state and should be given precedence in the nomination of neotypes (the kind of type to be nominated when the original types are lost).

Inaccessibility of types is another issue that needs to be addressed. The current wording of the ICNafp does not prevent deposition of types in private reference collections or in institutional reference collections that do not grant unrestricted access to their holdings. It would be preferable to stipulate that types must be available at the time names are published. Furthermore, if a type became unavailable, a substitute type could be nominated. In such situations, again, ex-type cultures present in multiple institutions would facilitate the nomination of new types when the original type is no longer available due to policies of the reference collection where the type is held or due to restrictions imposed by countries where the type has been collected (Yurkov et al. 2019; da Silva et al. 2023).

During the past 150 years, diagnostic tools for fungi evolved from very simple descriptions focused on morphology, distribution and host to a complex array of techniques. These now include physiological profiling, sequence-based DNA barcoding or phylogenetic reconstruction and phylogenomics (Libkind et al. 2020; Chethana et al. 2021; Lücking et al. 2021; Opulente et al. 2024). As a result, fungal taxonomy has seen a significant shift towards laboratory-based approaches (Boekhout et al. 2021; Lücking et al. 2021). This shift has led to the application of novel tools to old taxa, often relying on either original cultures or freshly re-collected specimens and cultures. The widespread adoption of laboratory-based techniques, including sophisticated microscopy and molecular-based identification techniques, resulted in a growing overlap of tools and approaches used in prokaryotic taxonomy and revoked some past discussions on the so-called “type cultures” of fungi.

In the *International Code of Nomenclature of Prokaryotes* (ICNP, formerly the *International Code of Nomenclature of Bacteria* (ICNB) or *Bacteriological Code*), the nomenclatural type is represented by a viable culture of the type strain that must be deposited in at least two publicly accessible culture collections in different countries from which subcultures must be available (Parker et al. 2019). This requirement of the ICNP strongly stimulated the development of cultivation and preservation techniques and the worldwide establishment of microbial culture collections. Many such collections have acquired the status of Biological Resource Centres (BRCs) and became certified and/or some accredited institutions following relevant biosafety, biosecurity, and management guidelines (Boundy-Mills et al. 2016). Application of professional management systems, standard operational procedures, and databases to keep the ever-growing number of records was essential to ensure that the collections’ holdings and the associated information will be properly safeguarded (Smith and Ryan 2012; Boundy-Mills et al. 2016; Reimer and Yurkov 2022).

Now, on the 60th anniversary since the ability to have living cultures as name-bearing types of fungi was ended, it is time to revisit this issue in view of the progress in cultivation and cryopreservation techniques, as well as dramatically improved skills and quality standards of culture collections. Yurkov et al. (2021) consequently advocated further steps towards liberalisation of the ICNafp rules concerning cultures as nomenclatural types, giving yeasts as an example. However, viable type material is important not only for yeasts or dimorphic fungi, but also for other cultivable filamentous fungi studied for their distinctive morphological structures (e.g., specific type of spores), growth properties, secondary metabolites, and fungal-host interactions. The examples below demonstrate how viable cultures may contribute to the advancement and stability of taxonomy within different fungal groups. We highlight the areas where the *Code*, both rules and community practices, may need improvement in managing material represented by viable cultures. We also revisit the requirements governing the deposition of living material under the ICNafp and ICNP, and present proposals that outline a roadmap for establishing a more transparent and user-friendly system for viable nomenclatural types under the Chapter F, the section of the ICNafp that includes provisions specific to names of fungi.

## The utility of viable fungal cultures

This section reviews the use of living cultures for taxonomic studies across diverse groups of fungi. With the examples below, we emphasize the necessity for the preservation of viable material for nomenclatural purposes.

### Yeasts

Several species concepts have been applied to describe and interpret yeast diversity (Boekhout et al. 2021). The presently used approach can be viewed as the successor of the Genetic Species Concept (GSC) that was based on advances of Phenotypic- (physiology), Biological- (mating compatibility), and Phylogenetic (DNA barcodes) species concepts (Boekhout et al. 2021). The pipeline for yeast characterization that is often referred to as a polyphasic approach includes an investigation of species’ growth requirements and lifecycle on different media, a number of physiological and biochemical tests, and sequences of a few DNA barcodes. Being optional for species descriptions, whole genome sequences are gaining in importance in species delimitation and identification of hybrids (Boekhout et al. 2021; Čadež et al. 2019, 2023; Gabaldón 2020; Passer et al. 2019; Libkind et al. 2020; Groenewald et al*.* 2023).

Yeasts were among the very first *Fungi* cultivated in pure culture. The oldest yeast type strains preserved in culture collections are from the nineteenth century. These strains were repeatedly studied and re-identified following the changes in identification approaches that introduced complex physiological tests, DNA and cell biochemical properties, and nucleotide sequences (Boekhout et al. 2021). This was only possible due to access to viable material. Similarly, revisions of generic concepts or resolving taxonomic synonyms heavily depend on a thorough investigation of revitalised reference material, “ex-type” cultures, which have sometimes been incorrectly called “type strains” in the literature (Yurkov et al. 2021). These carefully identified and characterised isolates, which were deposited in one or more culture collections helped to resolve and correct species synonymy and substantially improve high-rank classification in *Saccharomycotina* (Yurkov et al. 2021; Groenewald et al*.* 2023; Liu et al. 2024), *Agaricostilbomycetes* (Wang et al. 2015a), *Cystobasidiomycetes* (Wang et al. 2015a), *Microbotryomycetes* (Wang et al. 2015a), *Tremellomycetes* (Liu et al. 2015), and *Ustilaginomycotina* (Wang et al. 2015b). Had a single inviable specimen been used as holotype of a yeast species, no taxonomic reclassification would be possible with some modern approaches.

### Yeast states of dimorphic parasites

Some *Basidiomycota*, such as various lineages of jelly fungi and smuts (previously referred to as heterobasidiomycetous fungi), comprise dimorphic fungi and produce yeast states as part of their life cycle. These organisms usually alternate between an asexual unicellular yeast morph and a multicellular hyphal morph, in which often sexual reproduction takes place. Whereas the yeast morph is often considered to be saprobic, the hyphal morphs of the known species act as parasites, mostly of plants or other fungi (Aime et al. 2014; Begerow et al. 2014, 2017; Kruse et al. 2017; Schoutteten et al. 2023).

For most dimorphic heterobasidiomycetous fungi, solely the hyphal morph was originally characterised for species descriptions. The hyphal morph, usually coinciding with the sexual morph, comprises most of the characters used in conventional morphology-based taxonomy, but also provides essential information about the life cycle and ecology of these species. Physical specimens of the hyphal morphs allow identification of the host and/or substrate of the studied fungus. Only a few authors studying heterobasidiomycetous fungi additionally isolated, studied, and deposited the respective yeast morphs in order to provide authentic reference material for future molecular studies (e.g., Spirin et al. 2018; Schoutteten et al. 2023). When isolated and grown in pure culture, these yeast morphs allow easy and efficient generation of DNA sequence data, which is necessary to infer the evolutionary relationships of these fungi and assess species boundaries (Boekhout et al. 2021). Especially in the case of dimorphic (myco)parasites, separation of host and the fungal parasite is essential to perform molecular investigations with these fungi. Furthermore, isolation of the yeast morphs of dimorphic Basidiomycota allows to apply a polyphasic approach in species delineation, in which DNA sequence data, morphological observations obtained from the hyphal morph, and physiological data from the yeast morphs are combined.

Especially in *Tremellomycetes* (*Agaricomycotina*), a class comprising mainly dimorphic mycoparasites and lichenicolous fungi, hundreds of species were described over the last two centuries based on morphological characteristics of the hyphal morph only. Unfortunately, fungarium material is available for only a subset of these taxa, and most of the available material does not allow investigation of the relevant properties necessary for comparison with currently well-characterised species, such as DNA sequence data and physiological assimilation profiles. The availability of viable yeast strains of these *Tremellomycetes* would allow to resolve taxonomic problems in this class much more efficiently. A problematic trend is that dozens of new species of *Tremellomycetes* were described over the last 10 years, without considering the currently available names in this group, for which no cultures and DNA sequence data are available (e.g., Kachalkin et al. 2019; Li et al. 2020).

In smut fungi, the majority of which reside within *Ustilaginomycotina*, most species have been described based on combinations of morphological characteristics (e.g., teliospore size and ornamentation, sorus location) and host information (Vánky 2012). As teliospore traits are limited, specimens on closely related hosts were often combined into the same fungal species. Likewise, delimiting species on the same host based on morphological differences was and is also a common practice. This has led to a plethora of species names that only now, with molecular work, can be examined for their validity. Further complications arise, as molecular studies have also shown that the *Ustilaginomycotina* are not restricted to smut fungi, but that other ecologies are also common in the subphylum (e.g., Wang et al. 2015a, b). A surge of new species descriptions in *Ustilaginomycotina* without considering previous described species that do not have a culture or sequence data available has been the result. Some smut fungi (e.g., *Tilletia* spp, *Ustilago hordei*, and *U. scitamineaum*) are of agricultural importance. Detailed molecular, physiological and genetic studies that could inform on the ecology and evolution of plant-parasite interaction could be significantly fostered by the availability of viable yeast strains.

### Aquatic hyphomycetes

Aquatic hyphomycetes are important key players in the decomposition and conversion of leaf litter in lotic systems (Gessner et al. 2007; Hyde et al. 2016). Since the discovery of aquatic hyphomycetes about 80 years ago (e.g., Ingold 1942), more than 300 species of these fungi have been described. Generic concepts were largely based on spore morphology and mode of conidiogenesis (e.g., Gulis et al. 2020), but these characters do not necessarily correlate with their phylogeny (Baschien et al. 2013; Johnston et al. 2019). It has been demonstrated that the morphology of these fungi varies with cultivation conditions (Descals 2020). As a result, a proper revision of these fungi would require viable cultures to study their morphology, (e.g., sporulation in submerged culture) and DNA-based phylogeny.

Within the last decades many aquatic hyphomycetes have been described by three leading researchers, namely Ludmila Marvanová, Enrique Descals and John Webster. In addition to the type material, they often kept accompanying isolates of aquatic hyphomycetes in culture. However, viable cultures of these fungi are extremely rare in public culture collections. For example, out of 1500 cultures of aquatic hyphomycetes deposited by Marvanová and co-workers in the Czech Collection of Microorganisms (CCM), only 30 are publicly available (online catalogue of CCM assessed on 22.05.2024). The rarity of viable ex-type cultures often leads to taxonomic confusion, as in the study by Lombard et al. (2015) revising two genera, *Heliscus* and paraphyletic *Flagellospora*. The lack of sequence data from missing or unavailable ex-type cultures has a strong negative impact on environmental culture-independent studies resulting in false or no identification of key players in aquatic environments.

Type specimens prepared as permanent slides or dried are currently available from the Prague Museum (PR, Prague, Czech Republic), IMI Fungarium (K(M)-IMI, Kew, UK) and Madrid Botanical Garden (MA, Madrid, Spain) cannot help in the revision of genera of aquatic hyphomycetes. These specimens proved to be inviable in most cases, as did many authentic cultures preserved under mineral oil. Elaboration of modern generic concepts and delimitation of morphologically cryptic species of aquatic hyphomycetes is impossible without viable cultures (Baschien et al. 2006, 2013; Tsui et al. 2016). Likewise, a routine preservation of viable isolates is crucial for investigating genetic and morphological as well as biogeographic variation in aquatic fungi (Duarte et al. 2016; Johnston et al. 2019; Vasconcelos Rissi et al. 2023). The present situation in aquatic hyphomycetes is very unfortunate with many legitimate but unresolved species and genus names, which are difficult to revise due to the lack of viable material. The availability of strains is mandatory for future taxonomic work that must also include re-sampling as well as epi- and neotypification of earlier described fungi.

### Penicillium

For much of the history of *Penicillium*, its classification and identification have been based on morphology, which is notoriously difficult to interpret. Monographic treatments of the genus and its associated sexual morph (teleomorphic) genera *Eupenicillium* and *Talaromyces* were published by Thom (1930), Raper and Thom (1949), Pitt (1980) and Ramírez (1982). These works typically emphasised the need for standardised working methods to delineate and classify species more precisely. In the case of *Penicillium*, Pitt (1980) had to prepare dried cultures from living cultures in order to provide *Code*-compliant types for numerous previously described species and deposit the dried cultures in what is now K(M)-IMI.

In the early days of DNA sequencing and phylogenetic analyses, *Penicillium* was shown to be polyphyletic segregating into two clades defined by *Eupenicillium* and *Talaromyces* (Berbee et al. 1995; LoBuglio et al. 1993). Peterson (2000) then noted that the classification of subgenera based on the branching patterns of conidiophores did not agree with the phylogenetic structure. Frisvad and Samson (2004) subsequently revised and stabilised the taxonomy of *Penicillium* subgenus *Penicillium* by introducing the polyphasic species concept (or consilient concept of species), where species are characterised based on morphological data, extrolites (or secondary metabolites) and DNA sequence data. *Penicillium* taxonomists still recommend this polyphasic approach to define species (Houbraken et al. 2020; Visagie et al. 2023). Visagie et al. (2014) provided guidelines for working with and describing new species of the genus. This included details ranging from the growth media formulations and incubation conditions required for morphological comparisons to the primers, amplification conditions and gene regions required for phylogenetic comparisons. These guidelines were published along with an updated list of accepted species, which built on the earlier lists by Pitt and Samson (1993) and Pitt et al. (2000). The update was significant with species accepted only if a representative DNA sequence was available for them. This stems from the realisation that morphology has become largely uninformative and unreliable for identifications, not only because it is difficult to interpret, but also because the last monographs dealing with the genus were published more than 40 years ago. Each of the 354 accepted species was listed with the typical information published in such lists, but also included additional information such as MycoBank numbers, accessions to live ex-type cultures, subgenus classification and GenBank accession numbers for sequences obtained from ex-type cultures. These data paved the way for sequence-based identifications to become a reality for *Penicillium*. They also enabled easier discovery of new species, with Houbraken et al. (2020) accepting 482 *Penicillium* species and Visagie et al. (2023) accepting 535. *Penicillium* is a good example of how traditional dead specimens are not as useful as live cultures. To fulfil valid publication under ICNafp, a number of new names in *Penicillium* have been based on dried specimens (rather than metabolically inactive cultures) and such specimens do not represent the associated name as well as the living culture and its DNA sequences do.

### Xylariales

The aforementioned situation with the genus *Penicillium* is similar to that in many other groups of fungi, which have originally been described from their morphological characters and were later found to have a rather complex life cycle. A good example includes fungi in *Xylariales*, which were originally distinguished based on the morphology of their conspicuous stromata in the eighteenth and nineteenth century and details of the ascospores. *Xylariales* mostly form sporing structures and grow saprotrophically on woody substrates (Daranagama et al. 2018). Later, it was found that these fungi are among the most ubiquitous endophytes of seed plants and that some are closely associated with insects. Their current taxonomy is, as in *Penicillium*, based on a polyphasic approach, including morphological, chemotaxonomic, and molecular data (Helaly et al. 2018; Becker and Stadler 2021). Indeed, the type of conidiogenous structure is significant for assignment of these species to families, while the production of certain, rare secondary metabolites can be an important character at the genus level. Without the availability of living cultures, classification according to the current system is heavily reliant on multi-gene genealogies, and, in some instances, refined via phylogenomic studies that take into account the distribution of the genes encoding for secondary metabolites, becomes unfeasible (Wibberg et al. 2021; Kuhnert et al. 2021; Franco et al. 2022). The availability of protein-coding genes, which are nearly impossible to amplify by PCR from dried agar plates, is essential for species delimitation in this group of fungi because rDNA sequences have been shown to be unsuitable due to intragenomic polymorphisms and highly conserved nature (Cedeño-Sanchez et al. 2024). In a remarkably fortunate situation, an ex-type culture of *Induratia apiospora* was available from a public domain collection allowing for its study 35 years after discovery of the species (Cedeño-Sanchez et al. 2023) The availability of this viable culture for study helped to correct an error made just two years prior, which had resulted in the erection of a family to accommodate these fungi. These examples show the importance of making it nearly obligatory to deposit living cultures for all groups of fungi that are frequently encountered in environmental samples and do not possess conspicuous morphological characters or with complicated lifestyles.

### Other pleomorphic taxa

Pleomorphism is an important phenomenon occurring in some taxa. Describing the holomorph of pleomorphic taxa posed a significant challenge in traditional, morphology-based taxonomy, before dual nomenclature was abandoned in 2011. Cultivation and in-culture studies, especially from single ascospores, helped to establish links between morphs before application of DNA-based tools (e.g., *Botryohypoxylon* and its asexual morph *Iledon*, Samuels and Rogers 1986). Modern DNA sequence-based taxonomy provides a strong basis for linking different morphs from different environments and unconnected observations. Obtaining good DNA sequence data from the species of interest often involves cultivation and a number of purification steps that highlight the vital role of cultures for research.

Cultures of newly isolated pleomorphic taxa have been used to observe their alternative morphs in vitro. This has been a common practice among taxonomists who mainly focus on studies of sexually typified genera in *Pezizomycotina*. Some asexually typified species have been reported with more than one asexual morph in culture, for example *Dichomera* (Barber et al. 2005) and *Readeriella* (Crous et al. 2009) “synanamorphs”. These examples show the importance of culturing and maintaining fungi in culture to observe asexual morphs or synasexual morphs that cannot be observed in vivo. The safe preservation of sporulating cultures (including asexual or synasexual morphs produced in vitro) by using drying and/or cryopreservation techniques serves a valuable complement to non-viable material. Particularly for pleomorphic taxa, viable cultures are indispensable for long-term investigations of fungal life cycles and transitions between morphs that may not be immediately observable. Safely preserved viable cultures offer a nearly infinite source of genetically stable material for culture experiments that can be replicated under various conditions and in different laboratories. These cultures enhance material availability for educational purposes, ease the re-observation of morphological characteristics, facilitate recognition (or re-discovery) of fungi in subsequent studies and establish connections to advanced technologies for more in-depth studies.

## Practical considerations for preservation of viable material

The lack of universal protocols for maintaining, preserving and reviving cultivated fungi is probably the most critical limitation for the successful application of viable cultures in experimental and taxonomic works.

Formal requirements applied to the material in culture collections should not be more restrictive than that of specimens in fungaria. A dead specimen can potentially be used for sequencing (but see Kurtzman 2004), allowing it to be used for identification purposes. However, it is of limited use to gain any additional insights, including its physiology, interactions and reactions to abiotic and biotic stressors. Additionally, the risk of specimen loss due to invasive investigation techniques may prevent its authentication. This issue does not arise with metabolically inactive cultures that can be revived. There is always a chance of an accidental loss of any collection holdings irrespective of the type of material (Mega 2020), but negative consequences thereof can be mitigated by creating back-ups and appropriate documentation. The importance of safe preservation of cultures is acknowledged in the *Code* by recommending to deposit the material in at least two reliable repositories, reputable culture collections; the wording later exchanged for genetic resource collections (reviewed in Yurkov et al. 2021). Application of several preservation techniques, storage in spatially separated facilities, and exchange of cultures between culture collections further increase the chances to safeguard the material (Smith and Ryan 2012). These and other important recommendations have been accumulated in the best practice guidelines for Biological Resource Centres (BRCs) for safe preservation of living organisms published by the Organisation for Economic Development and Co-operation, OECD (Smith and Ryan 2012). These OECD guidelines were adopted by many BRCs becoming an integral part of common collection practices that include in particular internal quality control procedures to attest purity, viability and authenticity of the deposited material (Hawksworth and Schipper 1989; Santos and Lima 2001; Smith and Ryan 2008, 2012; Boundy-Mills et al. 2016; Yurkov et al. 2021).

Historic records in culture collections play an important role in validating the origin of strains and safeguarding against unintended replacements. Thereby, collections can also facilitate precise labelling of collection strains, indicating original and authentic material. In addition to the literature describing species, some collections provide additional information on strain authentication, including accession and identification date, as well as published quality control sequences. This creates a transparent system wherein users can access strains, their history (e.g., isolator, identifier, and depositor) and associated metadata through a collection catalogue.

Collections issue and supply depositors with deposition certificates which state that material has been received, checked, and preserved in the open collection following internal quality standards. This approach has been used for publication and validation of names of prokaryotes in the International Journal of Systematic and Evolutionary Microbiology (IJSEM) to prove that the type strains of prokaryotes are available according to the ICNP rules (Parker et al. 2019). Although not covered by the ICNP, the same requirement has been applied to new yeast taxa published by IJSEM. Considering the history of successful use of certificates of deposition, the requirement of Art. 40.8 to state that a culture is preserved in a metabolically inactive state (ICNafp Shenzhen *Code*; Turland et al. 2018) could become superfluous if replaced with a requirement for a certificate issued by a culture collection. Such a requirement could be built in at a later stage to the current process by which names of fungi are compulsorily registered prior to publication, in order to be valid (Art. F.5). Collections that utilise cryopreservation for their holdings systematically document their cultures (e.g., Reimer and Yurkov 2022). Unlike the currently mandated statement about preservation in a metabolically inactive state, a certificate issued by a culture collection is a more reliable source of information about preservation techniques as well as about viability and authenticity of the strain.

## Where the *Code* can be improved

Provisions of the *Code* require, for the valid description of a novel fungus, the designation of a single holotype, which can be a specimen, dried culture, slide preparation, illustration, or a metabolically inactive culture. Formally, the metabolically inactive culture can be viable, but it does not have to be. Subcultures of the nomenclatural types (ex-type cultures; sometimes incorrectly referred to as type cultures or strains in the literature) can be deposited in culture collections in the first place as isotypes or ex-type cultures, though there is no common practice for distinguishing isotypes (metabolically inactive duplicates prepared at the same time as the holotype) and ex-types (resuscitated from a metabolically inactive type) preserved in collections. The present system operates with a complex terminology, which can be confusing in the case of fungi growing in culture, like yeasts and some other fungi (Yurkov et al. 2021), and does not provide additional clarity on the kinds of material (e.g., isotype or ex-type culture) used in studies after the original description. This is because the ex-type cultures do not have any formal nomenclatural status under the current *Code*.

The abandonment of the dual nomenclature introduced an additional layer of complexity to the taxonomy of pleomorphic fungi. In some studies, living cultures of various morphs belonging to the same species, derived from non-viable specimens, are deposited as supplementary nomenclatural references. Examples include *Paraphaeosphaeria michotii* (Wanasinghe et al. 2018) and *Synnemasporella aculeans* (Fan et al. 2018). While it is common to designate all living cultures originating from the holotype as “ex-type cultures”, alternative morphs discovered later, for example through mating, also necessitate a distinct taxonomic treatment.

For fungi, the introduction of mandatory digital identifiers issued by designated nomenclatural repositories, at the Melbourne IBC in 2011 for names (McNeill et al. 2012: Art 42, now May et al. 2019: Art. F.5.1) and in 2019 for typification acts (May et al. 2019: Art. F.5.4) provides the basis for a transparent system to track the status of names and typification acts. Information that should be included as part of the registration process includes, for names, the name itself, but also the authorities and the place of publication and details of the type; and includes, for registration acts, the name, the author designating the type, and details of the relevant types. A strain identifier for a reference collection has a similar role as a fungarium accession number. A useful and technically feasible next step is to extend nomenclatural type information with details about ex-types, their progenies, or references to alternative morphs within a single electronic system.

We believe that mycologists working with different groups of fungi will benefit from the improvement of rules applied to viable holotypes and other material deposited for nomenclatural purposes under Chapter F. Nevertheless, we do not intend to make this practise binding for all mycologists. In our opinion, the community represented by ICTF taxonomic sub-commissions and working groups should decide whether it is technically feasible to recommend or perhaps even enforce viable holotypes as best practise for a specific group. When depositions of viable cultures as types are feasible, streamlining and enhancing the procedures for deposition could be achieved by consolidating these in ICNafp Chapter F. Below we present a roadmap with tasks to be achieved to establish a stable nomenclatural system that will properly manage taxa typified by viable specimens by defining the nomenclatural status of ex-type cultures under Chapter F.

### A roadmap for liberalisation of rules for metabolically inactive viable holotypes

1. While deposition of a holotype that is a specimen or a holotype that is a viable culture (in a metabolically inactive state) can be performed according to the current provisions of the *Code*, deposition of a viable ex-type culture with a nomenclatural meaning would require a few new rules under Chapter F. The current recommendation to deposit viable cultures in two culture collection is not binding, and the progeny of the viable holotype or isotype has no taxonomic status.
2. Managing nomenclatural records for viable specimens electronically, and following the newly suggested rules and procedures under Chapter F, can significantly enhance the transparency and accuracy of type material information. A further development of specimen or strain records in a nomenclatural repository can lead to a system that displays links between a holotype and its progenies (“ex-type” cultures deposited in other collections), authentication reports, and deposition certificates from collection curators. The availability of this information in a dynamic database may help to avoid incorrect records, at the time of publication, under the ICNafp Art. 9.2, including those under Art. 40.6, 40.7 and 40.8, which are not correctable after publication under the present wording of the ICNafp.
3. Proposed retroactive recognition of some “imperfect” descriptions that followed the presentation of the description of *Candida populi*, which has been used as the example of a metabolically inactive specimen in the *Code* Art. 8.4, Ex. 12 (but for which there was no statement in the protologue that the culture was lyophilized or preserved metabolically inactive in some other way). Explicitly, this means that, prior to 1 January 2019, species for which the type was known to be (or can reasonably be assumed to be) preserved in a metabolically inactive state but a statement of that was omitted or presented inconclusively, are not invalid under Art 40.8 (Turland et al. 2018). Clarity on this retroactivity avoids the need to validate potentially invalid names based on cultures, published prior to 1 January 2019. For names based on cultures published on or after 1 January 2019, the current requirement to state in the protologue that the culture is preserved metabolically inactive remains in force.
4. Article 40.7 requires that the single institution in which the holotype is lodged must be specified for names introduced after 1 January 1990 (Turland et al. 2018) which does make a clear cut between old practices (when a single institution was not indicated) and descriptions after that date (when this is not now allowed). Due to varying community practices used for cultivable fungi, the format for specification of types was often different to that for other organisms covered under the *Code*. A collaborative effort by the NCF, nomenclatural repositories, and ICTF subcommissions and working groups should determine acceptable formats of typification that were presented inconclusively in past descriptions. We have suggested some amendments to the *Code* to assist in these determinations.

## Proposals

The following proposals (in **bold** type) to amend Chapter F of the International Code of Nomenclature for algae, fungi, and plants (ICNafp or the *Code*) for a viable specimen to represent the nomenclatural type of a name are proposed.

**PROPOSALS**

Art. F5.5

For an identifier to be issued by a recognized repository as required by Art. F5.4, the minimum elements of information that must be accessioned by author(s) of type designations are the name being typified, the author designating the type, and those elements required by Art. 9.21, 9.22, and 9.23.

**The registration of a name for which the type is a viable metabolically inactive culture must include the kind of material (viable specimen), holotype and isotype designations (culture collection numbers) and the type of preservation (metabolically inactive state).**

**Add a new article to Chapter F, under a new section CULTURES AS TYPES** **:** **Article F.X.**

**Art. F.X.1.**

**“From 1 January 2025, for a fungus based on a living culture, the holotype strain and any isotype strains must be registered according to Art. F5.4, F5.5 to be acceptable as a nomenclatural type.”**

**Recommendation F.X.1**

**“From 1 January 2025, for a fungus based on a living culture, its viable progenies shall be preserved metabolically inactive in at least two different publicly accessible culture collections (Rec. 8B.1).”**

**Art. F.X.2**

**“In case the holotype and isotype material are not available anymore, the oldest authenticated, culture of an ex-type progeny preserved in a metabolically inactive state attains the status of a neotype.”**

**Art. F.X.3**

**“For names of fungi based on cultures that are preserved in a metabolically inactive state, any type of writing that explicitly indicates in a non-contradicting way that a single culture is the holotype is to be considered a valid typification (see Ex. X.1).”**

**Example F.X.1**

**“In the description of Metschnikowia hawaiiensis Lachance, Starmer and Phaff (Int. J. Syst. Bacteriol. 40: 416. 1990), the type of the name has been designated as the type strain UWO(PS) 87–2167.2 (= ATCC 76059 = CBS 7432). The type of Metschnikowia hawaiiensis UWO(PS) 87–2167.2 (original culture) has been permanently preserved in a metabolically inactive state in the American Type Culture Collection (ATCC 76059 is the holotype) and the collection of the Yeast Division of the Centraalbureau voor Schimmelcultures (CBS 7432 is the isotype).”**

**Example F.X.2**

**In the description of Cryptococcus vishniacii Vishniac & Hempfling (Int. J. Syst. Bacteriol. 29: 155. 1979), the type of the name has been designated as the type strain MTSW 304Y268 (= ATCC 36649). The type of Cryptococcus vishniacii MTSW 304Y268 (original culture) has been permanently preserved in a metabolically inactive state in the American Type Culture Collection (ATCC 36649 is the holotype).**

**Example F.X.3**

**“In the description of Cryptococcus bacillisporus Kwon-Chung & J.E. Benn. (Int. J. Syst. Bacteriol. 28: 618. 1978), the type of the name has been designated as the type strain ATCC 32608 (= CBS 6955). The type of Cryptococcus bacillisporus has been permanently preserved in a metabolically inactive state in the American Type Culture Collection (ATCC 32608 is the holotype). Its metabolically inactive duplicate has been permanently preserved in a metabolically inactive state in the collection of the Yeast Division of the Centraalbureau voor Schimmelcultures (CBS 6955 is the isotype).”**

**Note F.X.1**

**“Descriptions that used the writing format “strain designation A” [=original culture] (= “culture-collection B” [=holotype] = “culture-collection C” [=isotype]) are considered valid typifications, even where the kind of type is not specified in the original publication, provided that the preserved cultures are in a metabolically inactive state. Unless otherwise indicated, the first-mentioned metabolic inactive strain deposited in a public culture collection is recognized as the holotype.”**

**Note F.X.2**

**“Superscript T (** ^**T**^ **) used to indicate the nomenclatural type and its progenies in descriptions has no meaning in the Code. It does not substitute an indication of a nomenclatural type.”**

**Note F.X.3**

**“Specification of abbreviated forms the herbarium, collection, or institution can be given in an abbreviated form (Art. 40.7 Note 4). When not explicitly stated in the description, acronyms of culture collections can be determined according to data sources managed by the WFCC-MIRCEN World Data Center for Microorganisms. Any other culture identifiers are to be interpreted as strain designations.”**

**Recommendation F.X.2**

**“The recommended format for typification of names of fungi based on viable cultures is: Holotype CULTURE_COLLECTION XXXX, stored in a metabolically inactive state; isotype CULTURE_COLLECTION XXXX, stored in a metabolically inactive state; ex-holotype culture CULTURE_COLLECTION XXXX, CULTURE_COLLECTION XXXX.”**

**Art F.X.4**

**“Before 1 January 2019, when the preservation of a type of a fungus in a metabolically inactive state is not clearly stated in the original publication (Art. 40.8), this is treated as a correctable error not preventing valid publication of the name, provided there is evidence that a type was preserved in a metabolically inactive state by the specified culture collection prior to the publication of the protologue.”**

**Cross references in the body of the Code**

Appropriate cross references to the new material in Chapter F (should it be accepted) will need to be added to the body of the *Code*, specifically in Art. 8.4, which can be done editorially, and will not require any formal action.

Type specimens of names of taxa must be preserved permanently and may not be living organisms or cultures. Nevertheless, cultures of algae and fungi, if preserved in a metabolically inactive state (e.g., by lyophilization or deep-freezing to remain alive in that inactive state), are acceptable as types (see also Art. 40.8). **For further provisions relating to type specimens of fungi that are viable cultures preserved in a metabolically inactive state, see Art. X, Chapter F**.

Glossary: ex-type (ex typo), ex-holotype (ex holotypo), ex-isotype (ex isotypo), etc. A living isolate obtained from the type of a name when this is a culture permanently preserved in a metabolically inactive state (Rec. 8B.2). **Living cultures of organisms treated as fungi derived from the revitalisation of holotype or isotype metabolically inactive cultures are termed ex-type cultures**.
